# Carbon ion FLASH irradiation reduces acute skin toxicity compared with conventional dose rate irradiation

**DOI:** 10.1038/s41598-025-32014-w

**Published:** 2025-12-10

**Authors:** Yukari Yoshida, Hiromu Suda, Mutsumi Tashiro, Ken Yusa, Masao Nakao, Koichi Ando, Akihisa Takahashi, Tatsuya Ohno

**Affiliations:** 1 Gunma University Heavy Ion Medical Center, 3-39-22, Showa-machi, Maebashi, 371-8511 Gunma Japan; 2 Department of Radiation Oncology, Gunma University Graduate School of Medicine, 3-39-22, Showa-machi, Maebashi, 371-8511 Gunma Japan

**Keywords:** FLASH, Ultra-high dose rate, Radiation, Carbon-ion beams, Skin, Biophysics, Cancer, Medical research, Oncology, Physics

## Abstract

FLASH radiotherapy, defined by ultra-high dose rates exceeding 40 Gy/s, has shown potential for widening the therapeutic window due to its demonstrated normal tissue-sparing effects while maintaining tumor control with electrons, photons, and protons. However, limited biological data are available regarding its effects with heavy ions. This study aimed to investigate the biological effects of carbon-ion FLASH irradiation by assessing acute skin reactions in mice. C3H/He mice received irradiation to the right hind limb with 34–58 Gy of carbon-ion pencil beam scanning at the entrance region (dose-averaged LET: 13 keV/µm), delivered at either conventional dose rate (CONV: 0.047–0.051 Gy/s) or ultra-high dose rate (FLASH: 166.7–190.8 Gy/s). Skin reactions were evaluated every other day for up to 5 weeks to monitor early responses. The temporal patterns of skin response were similar between the CONV and FLASH groups. However, FLASH group consistently resulted in lower reaction scores across all doses and time points. Dose–response curve indicated a tissue-sparing effect with FLASH group, requiring approximately 1.5 times higher dose to produce the same biological effect as CONV group. These findings confirm the normal tissue-sparing potential of carbon-ion FLASH irradiation, with the FLASH effect quantitatively demonstrated through dose–response comparisons.

**Supplementary Information:**

The online version contains supplementary material available at 10.1038/s41598-025-32014-w.

## Introduction

Radiotherapy remains one of the most widely used and effective modalities for cancer treatment. Its success largely depends on widening the therapeutic window—maximizing tumor dose while minimizing exposure to surrounding normal tissues. Particle therapy using protons or carbon ions has gained attention due to superior dose conformality compared to photons. Notably, carbon ions offer a 2–3-fold higher antitumor efficacy than photons^1^, further enhancing the therapeutic window. However, even with advanced techniques like particle therapy, some tumors remain untreatable due to dose limitations caused by tumor radioresistance or proximity to radiosensitive organs. This underscores the need for new strategies to further expand the therapeutic window.

FLASH radiotherapy is an emerging technique that delivers radiation at ultra-high dose rates exceeding 40 Gy/s in a single session. Preclinical studies have shown that FLASH irradiation can reduce normal tissue toxicity while maintaining comparable tumor control. To date, research on FLASH has been conducted using mainly electrons, photons, and protons^2,3^. The phenomenon, known as the FLASH effect—a protective effect on normal tissue—has been observed across various models, including mice^4^, zebrafish embryos^5,6^, and mini pigs^7^, and in multiple organs, including the lung^8,9^, intestine^10–12^, brain^13–15^ and skin^16–20^. Tumor studies using orthotopic and xenograft models have shown that FLASH irradiation provides antitumor efficacy comparable to conventional dose rate (CONV) irradiation^7,8,21–23^, reinforcing its potential to reduce treatment-related toxicity and expand the therapeutic window. Although the clinical application of FLASH irradiation using carbon ions is highly anticipated, biological data on carbon-ion FLASH irradiation—particularly in vivo—remain scarce^24^.

This study aimed to assess the biological effects of carbon-ion FLASH irradiation using scanning pencil beam delivery in a mouse model, focusing on acute skin toxicity as a marker of normal tissue response.

## Materials and methods

### Animals

Female C3H/He mice (9 weeks old) were obtained from SLC Co., Ltd. (Shizuoka, Japan) and acclimated for 1 week before irradiation. The mice were bred and housed under specific pathogen-free (SPF) conditions and transferred to a conventional (non-SPF) facility following irradiation. Environmental conditions were maintained at a temperature of 22–24 °C with a 12-h light/dark cycle. Hair on the right hind limb was removed using a commercial depilatory cream (Shiseido Co., Ltd., Tokyo, Japan) 5 days before irradiation.

Sixty-four mice were used in this study, with eight animals assigned to each radiation dose group. All scoring and animal evaluations during the follow-up period were conducted in a blinded manner relative to the assigned treatment. All experiments adhered to the ARRIVE guidelines (Animal Research: Reporting of In Vivo Experiments). In addition, all animal procedures strictly followed the Guidelines for Laboratory Animal Management in Biomedical Research and were approved by the Animal Care and Experimentation Committee of Gunma University, Showa Campus (Approval No. 23 − 005).

### Irradiation

**Fig. 1:**
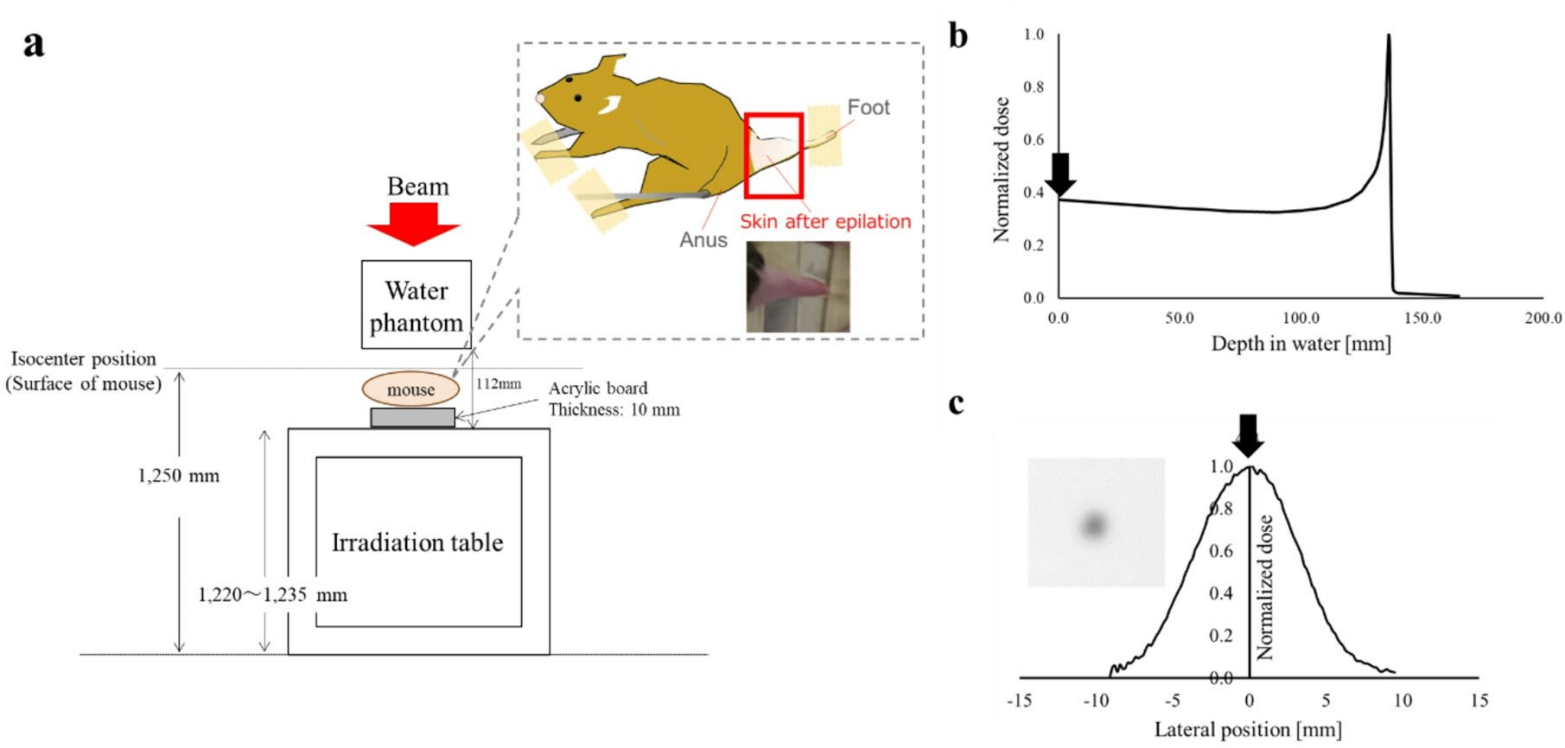
Irradiation setup and dose distributions. **(a)** Image showing the mouse irradiation setup. The recorded dose distributions in depth **(b)** and laterally **(c)** were measured using an ion chamber and films. The arrows indicate the positions of the skin surface of the mouse.

Irradiations were conducted using a 290 MeV/u ^12^C-ion vertical scanning beam at the Gunma University Heavy Ion Medical Center (GHMC)^25^. The dose-averaged linear energy transfer (LET_d_) was estimated to be 13 keV/µm using Monte Carlo simulations Geant4, corresponding to the entrance region of the Bragg peak of carbon-ion beams (Fig. 1b). The delivered beam profile at the isocenter (i.e., sample position) was measured using a radiochromic film (Fig. 1c). Technical details of the beamline have been previously described^26^. The distance of the beam spots in the scanning pattern was 2 mm, and the beam size was defined using a Gaussian profile with a standard deviation of 2.2 mm. The mice were anesthetized *via* intraperitoneal injection of ketamine (80 mg/kg) and xylazine (15 mg/kg) before irradiation. Local irradiation was administered to the right hind limb of each mouse, which was secured in a Lucite jig using tape (Fig. 1a). The lateral field size was 4 × 4 mm² (3 × 3 scanning spots), and all irradiations were delivered using a single two-dimensional scan. The field size was still sufficiently large to permit reliable observation of skin reactions, allowing meaningful comparisons between FLASH and CONV irradiation.

Single-fraction doses of 34, 42, 50, or 58 Gy were administered. Under FLASH conditions, the entire dose was delivered in a single beam spill at an intensity of approximately 1 × 10⁹ particles per second (pps), achieving an average dose rate exceeding 100 Gy/s (Table 1). For example, under the FLASH condition with a nominal dose of 58 Gy, the total irradiation time for the entire field within a single spill was 305 ms. Considering the 3 × 3 spot configuration and a beam size with a standard deviation of approximately 2.2 mm, the instantaneous dose rate at the center of each spot was estimated to be approximately 318 Gy/s. In contrast, for CONV irradiation, the beam intensity was reduced and doses were delivered over multiple spills at an average dose rate of 0.047–0.051 Gy/s. The average dose rate was calculated by dividing the target dose by the total irradiation time. The dose within the irradiation field was measured using an Advanced Markus chamber (PTW 34045) positioned at the field center. Preset dose values for each spot were calibrated to ensure that the measured dose matched the intended value, and all spots were assigned identical preset settings. For each irradiation condition, five measurements were performed to determine the mean dose and standard deviation.

**Table 1:** Measured dose and dose rate under each condition.

Setting	Nominal dose [Gy]	Dose [Gy]	Dose rate [Gy/s]
FLASH	34	33.8 ± 1.04	166.7 ± 1.7
	42	42.9 ± 2.84	184.9 ± 5.3
	50	50.4 ± 3.50	178.6 ± 6.3
	58	58.2 ± 2.54	190.8 ± 4.8
CONV	34	34.0 ± 0.08	0.051 ± 0.0000
	42	42.0 ± 0.12	0.051 ± 0.0001
	50	50.6 ± 0.73	0.047 ± 0.0003
	58	58.2 ± 0.18	0.047 ± 0.0001

For each setting, mean and standard deviation of five measurements are shown.

FLASH, ultra-high dose rate. CONV, conventional dose rate.

### Endpoints and data analysis

Skin reactions on the irradiated legs were evaluated every other day for up to 5 weeks. Reactions were scored using a previously established skin score table as described in^27^ (see Supplementary Table S1 online). Skin reactions were assessed by one researcher blinded to the study groups.

The dose–response curve for skin toxicity was fitted to the data using logistic analysis. Skin toxicity in each mouse was evaluated as the percentage of animals that reached each score within the observation period. From this analysis, the toxic dose producing a response in 50% of mice (TD_50_) and its 95% confidence interval were calculated. The dose-modification factor (DMF), representing the tissue-sparing effect of FLASH irradiation, was determined as the ratio of the FLASH TD_50_ to the CONV TD_50_. For the logistic analysis, additional data for lower doses were required, making it difficult to obtain an adequate fit using only the data generated in this study. Therefore, results for 18 Gy obtained from a separate experiment were included. The 18 Gy data were derived from an experiment performed using the same methods as this study but with a different irradiation field (14 × 14 mm^2^). Although that study used a larger field size, which generally produces a greater biological effect, the skin score for both CONV and FLASH irradiation was 0. Thus, inclusion of this result does not overestimate the findings of the present experiment.

### Histological analysis

Skin samples were collected from the mice at 20 days and 37 days post irradiation. Anesthesia was administered via intraperitoneal injection of ketamine and xylazine, consistent with the protocol used during irradiation. The harvested skin tissues were embedded in OCT Compound (Sakura Finetek Japan Co., Ltd., Tokyo, Japan), sectioned into 5-µm-thick slices, and stained with hematoxylin and eosin for histological analysis. Epidermis thickness were measured using ImageJ software (NIH, freeware imaging software) by researcher blinded to the study groups.

### Statistical analysis

All statistical analyses were conducted using GraphPad Prism 8 (GraphPad Software Inc., San Diego, CA, USA). Comparisons between groups were made using unpaired t-tests, as appropriate. *p*-values < 0.05, < 0.01, or < 0.0001 were used to denote statistical significance.

## Results

### Time course of FLASH and CONV irradiation in acute skin reactions

**Fig. 2:**
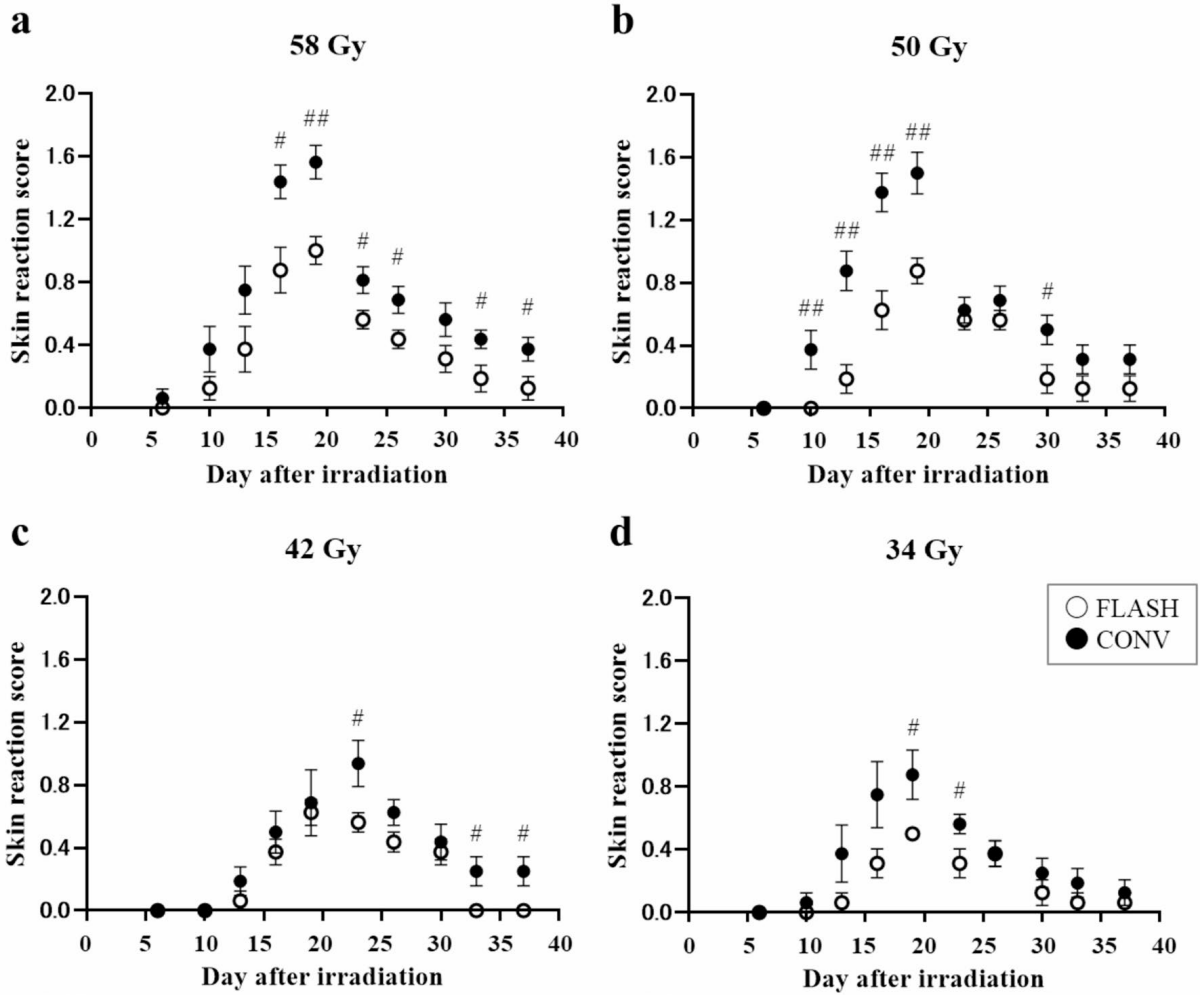
Time dependency of skin reaction in mice treated with ultra-high dose rate (FLASH) and conventional dose rate (CONV). **(a)** 58 Gy irradiated groups. **(b)** 50 Gy irradiated groups. **(c)** 42 Gy irradiated groups. **(d)** 34 Gy irradiated groups. For each dose, the points represent the mean and standard deviation of the skin reaction score (*n* = 8). # and ## indicates statistically significant difference in skin reaction score between FLASH and CONV at the same day after irradiation (#, *p* < 0.05. ##, *p* < 0.01). Bars represent the mean ± SE.

Skin reaction scores were plotted over time following either FLASH or CONV irradiation (Fig. 2). In all groups, skin reactions increased in a time-dependent manner, peaked, and then gradually declined, irrespective of dose rate. The peak skin reaction occurred at 19 days post irradiation for most groups, except for the 42 Gy CONV group, which peaked at day 23. Although the overall temporal patterns were similar between the FLASH and CONV groups, FLASH group consistently produced lower reaction scores across all doses and time points. The ratios of maximum skin reaction scores for CONV group compared with FLASH group were 1.56 at 58 Gy, 1.71 at 50 Gy, 1.50 at 42 Gy, and 1.75 at 34 Gy.

At 58 Gy and 42 Gy, significant differences between the CONV and FLASH groups were observed even at day 33 and 37, whereas no significant difference was found at 50 Gy. At day 37 post irradiation, the CONV group showed scores of 0.375 ± 0.08 at 58 Gy, 0.3125 ± 0.09 at 50 Gy, and 0.25 ± 0.09 at 42 Gy, indicating that the reaction scores decreased as the dose decreased. In the FLASH group, the scores were 0.125 ± 0.08 at 58 Gy, 0.125 ± 0.08 at 50 Gy, and 0 at 42 Gy. At 58 Gy and 50 Gy, two of eight mice in the FLASH group still exhibited a score of 0.5 on day 37. At 42 Gy, all eight mice had a score of 0. Therefore, although no statistically significant difference was detected, a difference in response was apparent between the CONV and FLASH groups at 50 Gy.

### Comparison of FLASH and CONV irradiation in acute skin reactions

All mice irradiated reached a score of 0.5 in both the CONV and FLASH groups (see Supplementary Figure S1 online). In the FLASH group, 37.5% of the mice irradiated with 58 Gy exhibited a score of 1.5, whereas no mice in the lower-dose groups (34, 42, and 50 Gy) did. In the CONV group, all mice irradiated with 58 Gy exhibited a score of 1.5, and 50% of those in the 34- and 42-Gy groups also reached that score. Furthermore, 25% of the mice irradiated with 58–50 Gy reached a skin score of 2.0 in the CONV group, whereas no mice irradiated with FLASH reached a score of 2.0 at any dose. These results suggest that the TD_50_ for FLASH irradiation may be higher than that for CONV irradiation at scores of 1.5 and 2.0, even though it was not possible to fit a reliable dose-response curve. Therefore, the DMF was calculated only from the data corresponding to a score of 1.0.

**Fig. 3:**
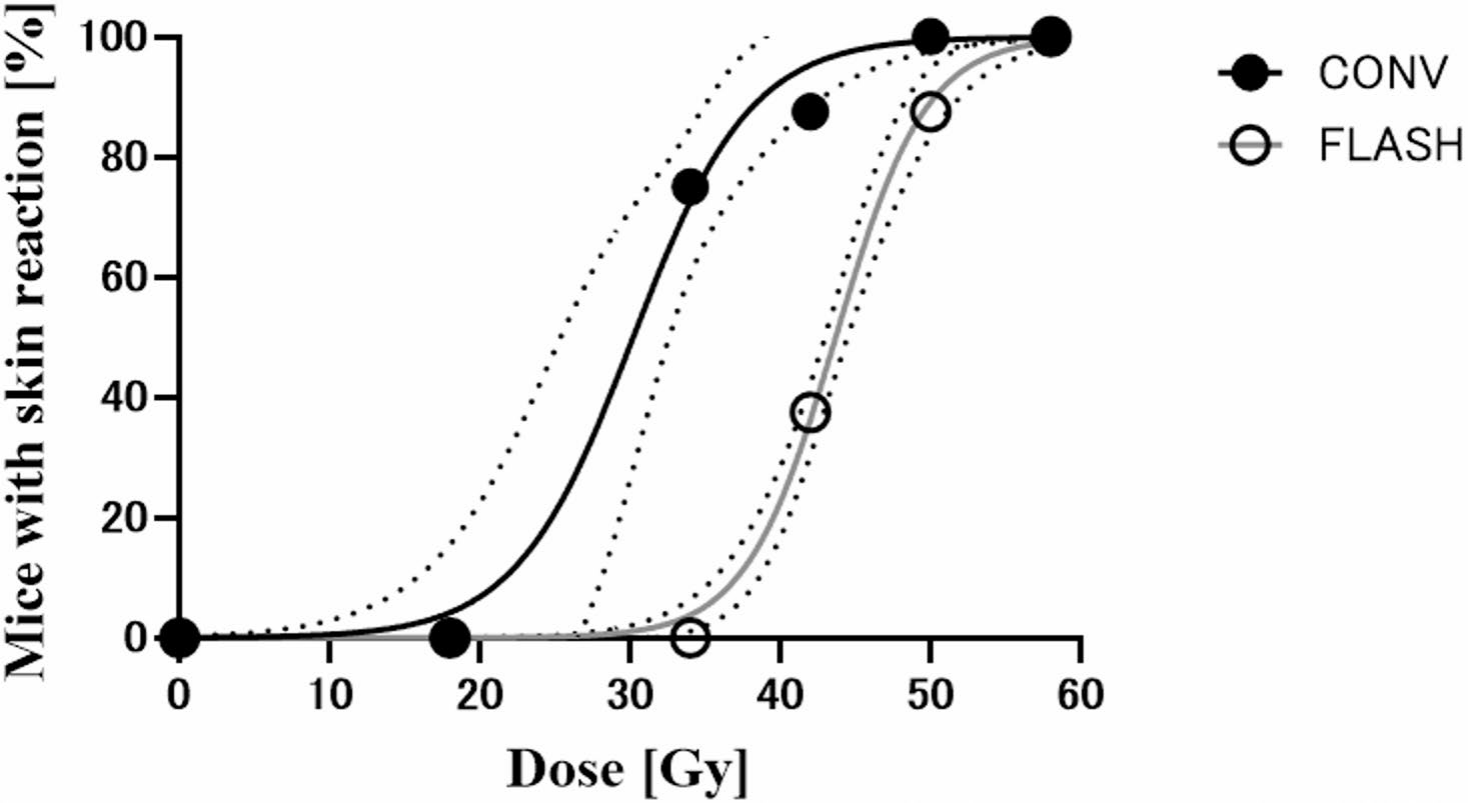
Dose dependency of mouse skin reaction. Percentage of mice in each dose group with skin reaction score 1.0 at either conventional dose rate (CONV) or ultra-high dose rate (FLASH) irradiation. CONV-irradiated group is indicated with closed circle and a black line. FLASH irradiated group is indicated with open circle and a gray line. The corresponding 95% CI are indicated dashed lines.

Figure 3 shows the dose–response curve for a score of 1.0. Dose–response curves were generated using the logit model, with data points representing the percentage of mice showing toxicity in each dose group. The dose–response curves for CONV and FLASH irradiation differed significantly (*p* < 0.0001), as demonstrated by their clearly separated trends and nonoverlapping 95% confidence intervals. The TD_50_ values with 95% confidence intervals were 30.2 (26.8–33.6) Gy for CONV irradiation and 43.7 (43.0–44.4) Gy for FLASH irradiation, resulting in a DMF of 1.45.

### Histological analysis of skin after FLASH and CONV irradiation

**Fig. 4:**
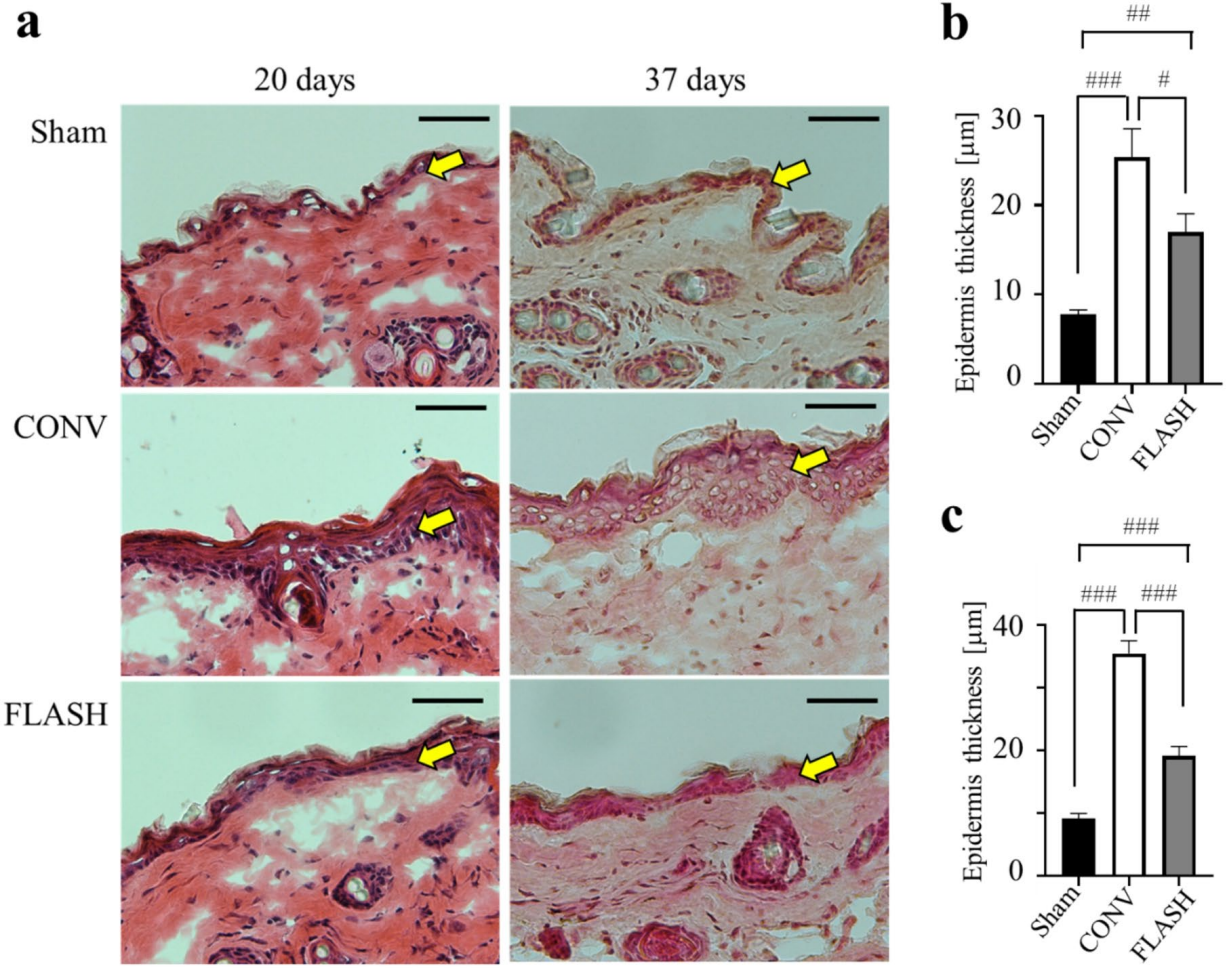
Histological analysis of skin after FLASH and CONV irradiation. **(a)** Representative hematoxylin and eosin images of the mouse skin in the nonirradiation (Sham), conventional dose rate irradiation (CONV), and ultra-high dose rate irradiation (FLASH) group at 20 days and 37 days after 58-Gy exposure. Scale bar is 50 μm. The yellow arrows indicate the epidermal layer. Histopathological evaluation of epidermal thickness was performed at 20 days **(b)** and 37 days **(c)** post irradiation (*n* = 3; statistical analysis using Welch’s t-test. #, *p* < 0.05; ##, *p* < 0.01; ###, *p* < 0.0001). Bars represent mean ± SE.

Histological analysis revealed marked epidermal thickening in the CONV irradiation with 58 Gy, whereas this response was notably reduced in the FLASH irradiation with the same dose (Fig. 4). Quantitative analyses were conducted at day 20 (the time point corresponding to the highest scores at high doses) and day 37 (when most mice had subsided damage and reached a score of 0; the endpoint of this study) post irradiation. Epidermal thickness in the CONV-irradiated skin was significantly increased at both day 20 (*p* < 0.0001) and day 37 (*p* < 0.0001) compared with the nonirradiated group. In the FLASH group, epidermal thickness was also significantly increased at day 20 (*p* = 0.0005) and day 37 (*p =* 0.0001) compared with the nonirradiated group. However, epidermal thickness after FLASH irradiation was significantly lower at both day 20 (*p* = 0.0322) and day 37 (*p* < 0.0001) than after CONV irradiation by the same dose.

## Discussion

In this study, we investigated the biological effects of carbon-ion FLASH irradiation on normal skin using a mouse model. Our findings demonstrated a clear normal tissue–sparing effect with FLASH irradiation. In particular, approximately 1.5 times higher doses were required with FLASH than with CONV carbon-ion irradiation to achieve the same biological response. This is the first study to quantitatively report the skin-sparing effect of carbon-ion FLASH irradiation *in vivo*.

The mouse skin reaction assay is a well-established system for evaluating radiation-induced effects, with early skin reactions serving as a classical and clearly defined endpoint in radiobiology. Skin erythema, ranging from mild redness to severe moist desquamation, has been thoroughly characterized^27^. The onset typically begins around day 10 post irradiation, progressing in a dose-dependent manner to peak severity. In present study, the temporal progression of skin reactions followed the expected pattern in both CONV and FLASH groups, with similar time-dependent trends observed in both groups.

Our results showed that DMF for FLASH irradiation was 1.45. A DMF larger than 1 suggests reduced toxicity for FLASH compared with CONV irradiation, confirming the presence of a FLASH-sparing effect. Sørensen et al. reported that a 44%–58% higher dose was required to induce equivalent acute skin damage in mouse legs using proton FLASH irradiation^20^. It is highly interesting that carbon ions produced the same effect as proton beams.

Minami et al. recently reported a sparing effect of carbon-ion FLASH irradiation at 6.5 Gy in an *in vitro* study using normal human dermal fibroblasts^28^. However, most studies reporting the FLASH effect on skin toxicity in mouse models have demonstrated this phenomenon at irradiation doses exceeding 30 Gy^17–20,23^. A distinctive feature of skin reactions to radiation is their dependence on field size. For example, smaller irradiated skin areas are more resistant to higher doses than larger areas.

A previous study by Ando et al. examined early skin reactions to carbon-ion beams in female C3H/He mice using conventional dose rate^29^. They reported skin reaction scores of approximately 4 at ~ 60 Gy and 1.5 at ~ 20 Gy using carbon-ion beams with a LET of 14 keV/µm. We predicted that the results of this study would show equal or lower toxicity than those reported by Ando et al., because the irradiation field size in our experiment was smaller than that used by Ando et al.; irradiate whole leg length. Therefore, we selected a dose range of 34–58 Gy for this investigation. In scanning irradiation, each point within the target is sequentially exposed to a narrow pencil beam that delivers the prescribed dose, resulting in a time-varying dose rate across the target. Moreover, the dose rate may fluctuate between spills. To simplify the definition and control of FLASH irradiation in this study, all samples were irradiated using a single spill. Because of the inverse relationship among field size, dose, and dose rate, reducing the irradiation field was necessary to achieve the dose and dose rate conditions required for this FLASH experiment. These physical limitations must be overcome to enable more detailed biological analyses and support the clinical application of FLASH radiotherapy in the future.

Radiation exposure can result in either increased or decreased epidermal thickness, depending on factors such as radiation type, dose, and duration. A common late side effect of radiotherapy is skin thickening, often attributed to radiation-induced inflammation and subsequent fibrosis in the skin and underlying tissues^30^. In the early phase, cell proliferation is temporarily inhibited in a dose-dependent manner, particularly affecting radiation-sensitive proliferating cells. A few days after radiation-induced tissue damage, the increased loss of stem and progenitor cells triggers enhanced proliferation of basal cells to restore skin tissue homeostasis^31^. Thus, epidermal hyperplasia, or excessive cell growth, represents a regenerative response during the healing process as the skin attempts to replace damaged cells. Therefore, early stage hyperplasia is also important for evaluating the extent of skin damage repair. Numerous previous studies have reported early epidermal hyperplasia. For example, a FLASH study using proton beams observed hyperplasia at 27 days post irradiation^19^. That study concluded that FLASH irradiation significantly reduced epidermal hyperplasia relative to CONV irradiation and favorably altered the pathology of wound healing compared with CONV irradiation.

In the present study, we observed epidermal thickening following CONV irradiation, whereas FLASH irradiation appeared to mitigate this effect. These data suggest that FLASH irradiation favorably alters the pathology of wound healing compared with CONV irradiation. The histological differences between CONV and FLASH irradiation suggest underlying variations in biological and molecular responses in these radiations.

To investigate the relationship between inflammation and skin reaction, we measured levels of transforming growth factor-beta1 (TGF-β1), inflammation marker, at 96 h post irradiation using enzyme-linked immunosorbent assay. However, no significant changes in TGF-β1 levels were detected in relation to skin reactions (see Supplementary Figure S2 online). The relation with FLASH effect on skin and TGF-β1 has been previously demonstrated with proton beam. Cunningham et al. assessed skin toxicity, leg contracture, and TGF-β1 levels in both plasma and skin and found reduced effects across all parameters in the FLASH group compared to the CONV group^18^. In that study, a much larger area of mouse skin was irradiated than in our experiment. Rudigkeit et al. reported that ear swelling and inflammation scores after FLASH irradiation were reduced compared with CONV irradiation in a mouse ear skin model^32^. However, no differences in TGF-β1 levels were observed between the sham and irradiated groups or between the CONV and FLASH groups. In their experiment, a 6.5 × 6.5 mm field was irradiated using proton beams. The authors discussed that a sufficient blood volume must be irradiated to detect measurable changes of TGF-β1 levels in the blood. It may therefore require irradiation of a larger blood volume (i.e., a larger field size) to observe a detectable change in TGF-β1 concentration.

To date, only two in vivo studies have reported on the effects of carbon-ion FLASH irradiation. Both investigations, conducted by the same research group, examined the biological differences between FLASH and CONV irradiation by assessing normal muscle tissue damage and antitumor responses in C3H/He mice with limb-implanted tumors. Regarding the impact on normal tissues, histological analyses revealed morphological disorganization of muscle fibers following both CONV and FLASH irradiation; however, FLASH irradiation induced less structural disruption in muscle tissue than CONV irradiation^33^. Furthermore, FLASH treatment resulted in significantly reduced collagen deposition, indicating less fibrosis formation relative to CONV irradiation^34^. These findings, together with the results of the present study, support the notion that carbon-ion FLASH irradiation exhibits a tissue-sparing effect similar to that observed with other radiation modalities. These promising outcomes highlight the need for further investigation into the protective effects of carbon-ion FLASH irradiation across various normal tissues and organ systems.

Given that the central premise of FLASH radiotherapy lies in its differential effects on tumor versus normal tissues, it is also essential to establish the impact of carbon-ion FLASH irradiation on tumor response. For clinical translation, it must be confirmed that carbon-ion FLASH irradiation does not compromise antitumor efficacy compared to CONV irradiation. Previous studies have reported enhanced tumor growth delay with carbon-ion FLASH irradiation relative to CONV irradiation in xenograft models^33,34^, indicating maintained—or even superior—cytotoxic effects against tumors. These findings suggest that carbon-ion FLASH irradiation enhances the therapeutic ratio of carbon-ion radiotherapy, highlighting its significant potential for clinical application.

## Supplementary Information

Below is the link to the electronic supplementary material.

Supplementary Material 1

## Data Availability

The datasets used and analyzed during the current study available from the corresponding author on reasonable request.
