# Supplementary material for: Carbon ion FLASH irradiation reduces acute skin toxicity compared with conventional dose rate irradiation
Source: Sci Rep. 2025 Dec 10;16:2307. doi: 10.1038/s41598-025-32014-w (PMC12816662; doi:10.1038/s41598-025-32014-w)
Supplement: Supplementary file 1 — Supplementary Material 1 [file 41598_2025_32014_MOESM1_ESM.docx]

**Supplementary Information**

**Carbon-ion FLASH Irradiation Reduces Acute Skin Toxicity Compared with Conventional Dose Rate Irradiation**

YUKARI YOSHIDA^1*^, HIROMU SUDA^1^, MUTSUMI TASHIRO^1^, KEN YUSA^1^, MASAO NAKAO^1^, KOICHI ANDO^1^, AKIHISA TAKAHASHI^1^, and TATSUYA OHNO^1,2^

^1^Gunma University Heavy Ion Medical Center, 3-39-22, Showa-machi, Maebashi, Gunma, 371-8511, Japan.

^2^Department of Radiation Oncology, Gunma University Graduate School of Medicine, 3-39-22, Showa-machi, Maebashi, Gunma, 371-8511, Japan.

*To whom correspondence should be addressed.

E-mail: [yyukari@gunma-u.ac.jp](mailto:yyukari@gunma-u.ac.jp)

This file includes:

Materials and methods for TGF-β1 level quantification

Table S1

Figures S1 and S2

**Materials and Methods for TGF-β1 level quantification**

A volume of 500–600 μL of blood was collected from the hearts of mice (n = 4 per group) anesthetized with ketamine/xylazine, 96 hours after CONV or FLASH irradiation into blood collection and separation tubes (Fuchigami, Kyoto, Japan). Serum was obtained via centrifugation at 3500 rpm for 5 minutes and stored at −80°C until measurement. TGF-β1 levels were quantified using a mouse TGF-β1 ELISA kit (Proteintech, IL, USA) according to the manufacturer’s instructions. Absorbance was measured at 450 nm using a MULTISKAN FC spectrophotometer (Thermo Fisher Scientific, MA, USA).

**Table S1.** Skin reaction score

Score

0.5

1.0

1.5

2.0

2.5

3.0

3.5

4.0

4.5

5.0

A: area of interest

Developing stage

doubtful difference from normal appearance

slight reddening

definite reddening

severe reddening

or definite dry desquamation

severe dry desquamation

slight moist desquamation (A ≦ 1/3)

definite moist desquamation (A ≦ 1/3) or

slight moist desquamation (1/3 < A < 2/3)

severe moist desquamation (A ≦ 1/3) or

definite moist desquamation (1/3 < A < 2/3) or

slight moist desquamation (A ≧ 2/3)

severe moist desquamation (1/3 < A < 2/3) or

definite moist desquamation (A ≧ 2/3)

severe moist desquamation (A ≧ 2/3)

Decaying stage

hair graying (A ≧ 1/2)

hair graying (A < 1/2)

no hair and thick skin

no scab and thin skin

scab (A ≦ 1/3)

scab (1/3 < A < 2/3)

scab (A ≧ 2/3)


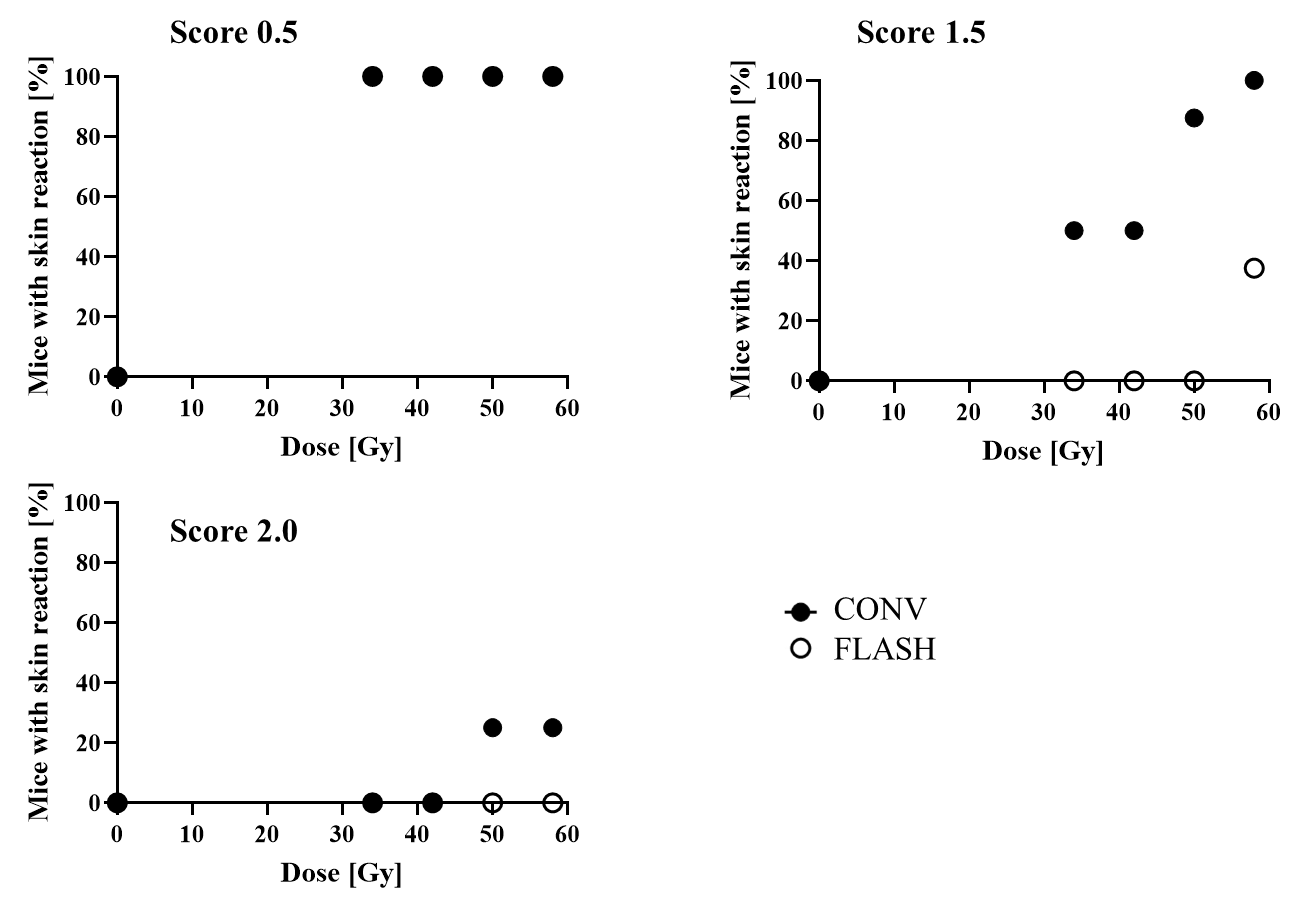


Figure S1. Dose dependency of mouse skin reactions. The percentage of mice in each dose group showing skin reaction scores of 0.5, 1.5, or 2.0 under conventional dose rate (CONV) or ultra-high dose rate (FLASH) irradiation. The CONV-irradiated group is indicated by closed circles with a black line, and the FLASH-irradiated group is indicated by open circles.


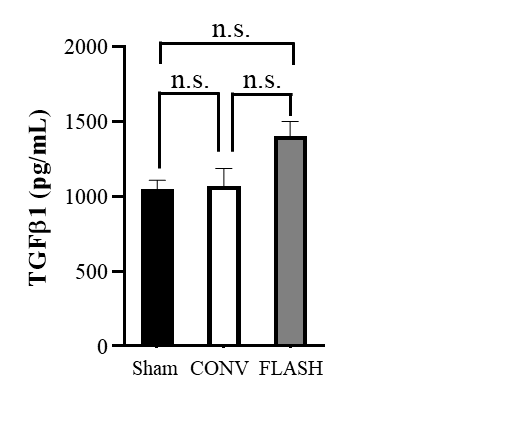


Figure S2. TGF-β1 expression in mouse skin from the nonirradiated (Sham), conventional dose rate (CONV), and ultra-high dose rate (FLASH) groups at 96 hours after 58 Gy exposure. Plasma levels of total TGF-β1 were measured using ELISA (n = 4 per group; statistical analysis using Welch’s *t*-test; n.s., not significant). Bars represent the mean ± SE.
